# Three-dimensional localization of point acoustic sources using a planar microphone array combined with beamforming

**DOI:** 10.1098/rsos.181407

**Published:** 2018-12-05

**Authors:** Hao Ding, Yumei Bao, Qi Huang, Chunxiao Li, Guozhong Chai

**Affiliations:** 1 Zhejiang University of Technology, Hangzhou, Zhejiang Province, People's Republic of China; 2 Zhijiang College of Zhejiang University of Technology, Hangzhou, Zhejiang Province, People's Republic of China

**Keywords:** spherical wave, acoustic imaging, beamforming, planar microphone array, sound source depth, 3D sound source localization

## Abstract

This paper presents a beamforming-based acoustic imaging (BBAI) method employing a two-dimensional (2D) microphone array that not only can locate an acoustic source in the *XY* plane parallel to the array, but can also identify the distance between the source and array in the *Z* direction, denoted as the source depth, and thus provides three-dimensional (3D) localization ability. In this method, the acoustic field is reconstructed on virtual *XY* planes at different distances along the *Z* direction. The source depth is then determined according to the virtual plane providing the maximum response of the acoustic field. The location of the source in the *X* and *Y* directions of the identified virtual plane can then be easily determined based on the standard beamforming principles of a planar array. The proposed BBAI method is evaluated based on simulations involving single- and multiple-point sources, and corresponding experimental evaluations are similarly conducted in an anechoic chamber. Both simulation and experimental results demonstrate that the proposed method is capable of locating acoustic sources in 3D space.

## 1. Introduction

Beamforming [1,2] is an advanced acoustic imaging technique that has been applied effectively for localizing and identifying acoustic sources on moving objects [3], high-speed trains and civil aircrafts in aeroacoustics [4–6]. At present, beamforming methods employing microphone arrays combined with signal processing technology have been widely used for both two-dimensional (2D) and three-dimensional (3D) acoustic-source localization in numerous fields [7–9]. In this paper, layer-by-layer scanning of the sound source field is achieved and thus realizes 3D acoustic-source localization and 3D sound source image output.

Beamforming methods have been extensively applied for 2D localization using planar microphone arrays, which can locate an acoustic source in the *XY* plane parallel to the array, but cannot identify the distance between the source and the array in the *Z* direction, denoted as the source depth. For example, generalized cross-correlation (GCC) beamforming has obtained precise 2D acoustic-source localization results in the time-domain [10]. Similarly, chirp Z transform (CZT) digital beamforming has been proposed for far-field acoustic-source localization in the frequency domain [11]. This method was demonstrated to overcome typical problems affecting other frequency-domain beamforming techniques such as zero-padded fast Fourier transform beamforming. In particular, the accelerated proximal gradient singular value thresholding-based linearly constrained singular canceler (APG-LCSC) algorithm [12] has been demonstrated to provide highly accurate 2D beamforming using a sparse array.

In 3D beamforming methods based on 3D microphone arrays, the concept of spherical harmonics has been employed with a spherical microphone array [13], and GCC has been employed with a polyhedral microphone array [14] for near-field reconstruction. Deconvolution based on spherical harmonics [15] and functional delay and sum (FDAS) [16] beamforming methods with spherical arrays have been shown to provide good spatial resolution and low sidelobes in the near-field. Moreover, FDAS with ridge detection (RD) and FDAS with RD and a deconvolution approach for the mapping of acoustic sources (DAMAS) [17] realized rapid acoustic-source localization as well as high resolution. Similarly, both generalized inverse beamforming (GIB) [18] and functional GIB (FGIB) [19] exhibited these characteristics using a double-layer microphone array.

In terms of acoustic sources, monopole and dipole sources are typically of great interest in aeroacoustics. For dipole sources, high-quality source maps have been established using orthogonally aligned planar microphone arrays [11,20,21] and non-planar microphone arrays [21]. For monopole sources, a planar-phased array [22] has provided good resolution for 3D acoustic imaging with Fourier deconvolution in the near field.

According to the above discussion, previous beamforming methods employing planar microphone arrays have mainly focused on acoustic-source localization on a 2D surface. While these methods provide an acoustic field hologram, they cannot determine the source depth, so they are inappropriate for 3D source localization [1,23–29]. However, present applications are increasingly concerned with acoustic sources located on the surfaces of complex objects or on complicated structures in 3D space. Yet, research regarding 3D acoustic-source localization remains relatively rare, and beamforming methods employing 3D microphone arrays remain limited to near-field reconstruction. And also compared with the 3D microphone array [30], using the 2D planar array in this study which has been commercialized can also achieve three-dimensional recognition ability with greater adaptability. Furthermore, quantitative analyses of localization error and the influence of frequency have been rarely investigated [31,32].

Deconvolution algorithms [33,34], especially the DAMAS algorithm [35], are the main methods used in recent years and provide high precision that cannot be achieved by traditional beamforming algorithms. But, there is a new problem of deconvolution algorithm, as the misleading point replaces the position of the continuous point distribution [36]. And its inevitable iterations result in much more computational complexity than traditional beamforming. For this problem, some scholars abandoned the deconvolution algorithm, returned to the traditional beamforming algorithm and proposed some advanced beamforming algorithms, for example, orthogonal beamforming [37], robustness adaptive beamforming [38] and functional beamforming [39]. In this paper, the 3D recognition ability of the traditional beamforming algorithm is realized with its faster calculation speed, which is different from the 3D recognition of the deconvolution algorithm [40,41].

To address these issues, this paper presents a beamforming-based acoustic imaging (BBAI) method employing a planar microphone array for the localization of point sources, which are similar to monopole sources. In the proposed method, the acoustic field is reconstructed on virtual *XY* planes at different distances along the *Z* direction. The source depth is then determined according to the virtual plane providing the maximum response of the acoustic field. The location of the source in the *X* and *Y* directions of the identified virtual plane can then be easily determined based on the standard beamforming principles of a planar array. As such, the proposed method not only can locate an acoustic source in the 2D *XY* plane parallel to the array, but can also determine the source depth, and thus provides 3D localization ability. The localization error and the influence of frequency of the proposed BBAI method are quantitatively evaluated by simulations and corresponding experiments in an anechoic chamber involving single- and multiple-point sources and a planar microphone array in the form of a 60-channel Brüel & Kjær WA-1558 sliced wheel array.

## 2. Experimental method

### 2.1. Three-dimensional localization of acoustic point sources using the BBAI method

**Figure 1.**
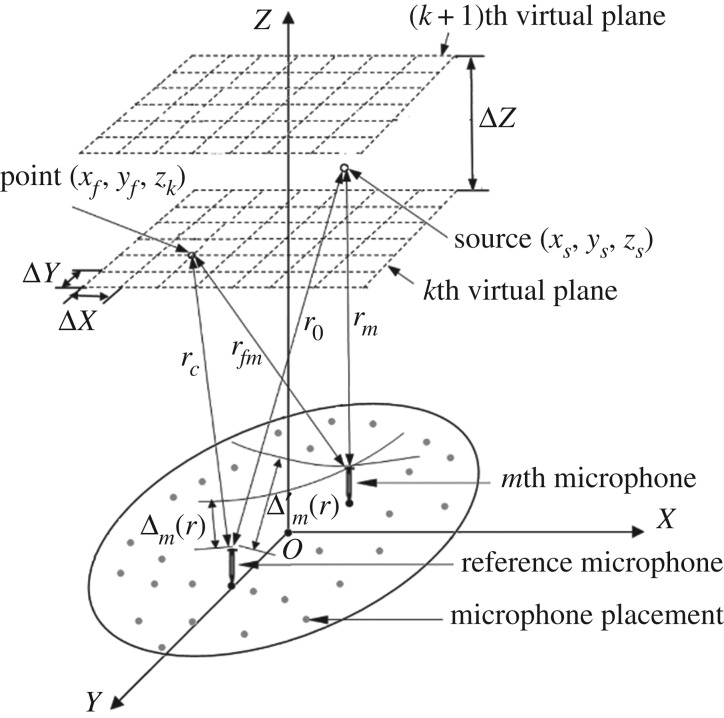
Three-dimensional localization of an acoustic source using the BBAI method.

As shown in figure 1, the BBAI method reconstructs the entire 3D acoustic field on virtual planes perpendicular to the *Z* axis at different distances according to the spherical wave hypothesis. The virtual plane spacing is defined as Δ*Z*, a meshed virtual plane is defined as a reconstruction plane, a mesh node is defined as a reconstruction point and the spacing intervals of adjacent points along the *X* and *Y* axes of an equivalent reconstruction plane are defined as Δ*X* and Δ*Y*, respectively. Acoustic field reconstruction is conducted by calculating the normalized beamforming power output at all reconstruction points, which is also referred to as the acoustic field response. Based on the spherical wave hypothesis, the distance a wave travels between an acoustic source located at (*x_s_*, *y_s_*, *z_s_*) and a microphone is equal to the distance between (*x_s_*, *y_s_*, *z_s_*) and that microphone. Here, we assume a planar microphone array consisting of a total of *M* microphones with coordinates (*x_m_*, *y_m_*, *z_m_*), *m* = 1, 2, … , *M*. Then, we designate the microphone denoted by *m* = 1 as the reference microphone with coordinates (*x*_1_, *y*_1,_ *z*_1_). The signal pressure received from the reference microphone *P*_1_(*ω*) can be defined as a function of the angular frequency *ω* of the source as follows [1]:

2.1 $$P_{1}(\omega )=\frac{P_{0}}{r_{1}}e^{\left(-jnr_{1}\right)}.$$

Here, *P*_0_ is the source strength, $r_{1}=\sqrt{{\left(x_{s}-x_{1}\right)}^{2}+{\left(y_{s}^{}-y_{1}\right)}^{2}+{\left(z_{s}-z_{1}\right)}^{2}}$ is the distance between the reference microphone and the source and *n* *=* *ω*/*c* is the wave number, where *c* is the propagation velocity of sound. In addition, we define $\Delta_{m}^{\prime }(r)$ as the delay in the wave arrival times between the reference microphone and the *m*th microphone, and is given as follows:

2.2 $$\begin{array}{rl} \Delta_{m}(r) & =r_{1}-r_{m}\\ \;\mathrm{and}\;{\Delta^{\prime }}_{m}(r) & =\Delta_{m}\frac{\left(r\right)}{c}. \end{array}\}$$

Here, $r_{m}=\sqrt{{\left(x_{s}-x_{m}\right)}^{2}+{\left(y_{s}-y_{m}\right)}^{2}+{\left(z_{s}-z_{m}\right)}^{2}}$ is the distance between the *m*th microphone and the source. Because a spherical sound wave is assumed to be radiated by the source, and the planar microphone array is far away from the source, the pressure signal received at the *m*th microphone (*P_m_*(*ω*)) will undergo attenuation relative to *P*_1_(*ω*), which can be expressed as follows [1]:

2.3 $$P_{m}(\omega )=P_{1}(\omega )\frac{r_{1}}{r_{m}}e^{\;j\omega \cdot {\Delta^{\prime }}_{m}(r)}.$$

The values of *P_m_*(*ω*) are then employed to reconstruct the *f*th reconstruction point (*x_f_*, *y_f_*, *z_k_*) on the *k*th reconstruction plane, *k* = 1, 2, … , *K*, where *K* represents the total number of reconstruction planes. First, we define the time delay $\Delta_{m}(r)$ for signals associated with the distance *r_c_* between (*x_f_*, *y_f_*, *z_k_*) and (*x*_1_, *y*_1_, *z*_1_) and the distance *r_fm_* between (*x_f_*, *y_f_*, *z_k_*) and (*x_m_*, *y_m_*, *z_m_*) as follows:

2.4 $$\begin{array}{rl} \Delta_{\;fm}(r) & =r_{c}-r_{\;fm}\\ \;\mathrm{and}\;{\Delta^{\prime }}_{\;fm}(r) & =\Delta_{\;fm}\frac{\left(r\right)}{c}, \end{array}\}$$

where $r_{c}=\sqrt{{\left(x_{f}-x_{1}\right)}^{2}+{\left(y_{f}-y_{1}\right)}^{2}+{\left(z_{k}-z_{1}\right)}^{2}}$ and $r_{\;fm}=\sqrt{{\left(x_{f}-x_{m}\right)}^{2}+{\left(y_{f}-y_{m}\right)}^{2}+{\left(z_{k}-z_{m}\right)}^{2}}$. According to the principle of delay and sum, the complex normalized beamforming pressure output *B*(*r*,*ω*) relative to the actual output on reconstruction point *f* is given as follows [1]:

2.5 $$B(r,\omega )=\frac{1}{M}\sum_{m=1}^{M}g_{m}P_{m}(\omega )e^{-j\omega \cdot \Delta_{m}(r)}=\frac{r_{1}}{M}P_{1}(\omega )\sum_{m=1}^{M}g_{m}\frac{1}{r_{m}}e^{\;j\omega \cdot ({\Delta^{\prime }}_{m}(r)-\Delta_{m}(r))}.$$

Here, *g_m_* is the weighting coefficient of microphone *m*. According to the triangle inequality in complex form [29], the normalized beamforming power output is obtained from equation (2.5) as follows:

2.6 $$|B(r,\omega ){|}^{2}={\left(\frac{r_{1}}{M}\left|P_{1}(\omega )\sum_{m=1}^{M}g_{m}\frac{1}{r_{m}}e^{\;j\omega \cdot ({\Delta^{\prime }}_{m}(r)-\Delta_{m}(r))}\right|\right)}^{2}.$$

From equation (2.6), $|B(r,\omega ){|}^{2}$ will be a maximum, which, in this paper, is denoted as $|B(r,\omega ){|}_{\max}^{2}$, only if the following condition is met:

2.7 $$\Delta_{1}^{\prime }(r)-\Delta_{1}(r)=\Delta_{2}^{\prime }(r)-\Delta_{2}(r)=\cdots =\Delta_{m}^{\prime }(r)-\Delta_{m}(r).$$

The values of $|B(r,\omega ){|}_{\max}^{2}$ are compared for all virtual reconstruction planes, and the position of the plane with the largest value along the *Z* direction represents the source depth. The source location in the *X* and *Y* directions can then be easily identified based on standard beamforming principles.

### 2.2. Simulation procedure

**Figure 2.**
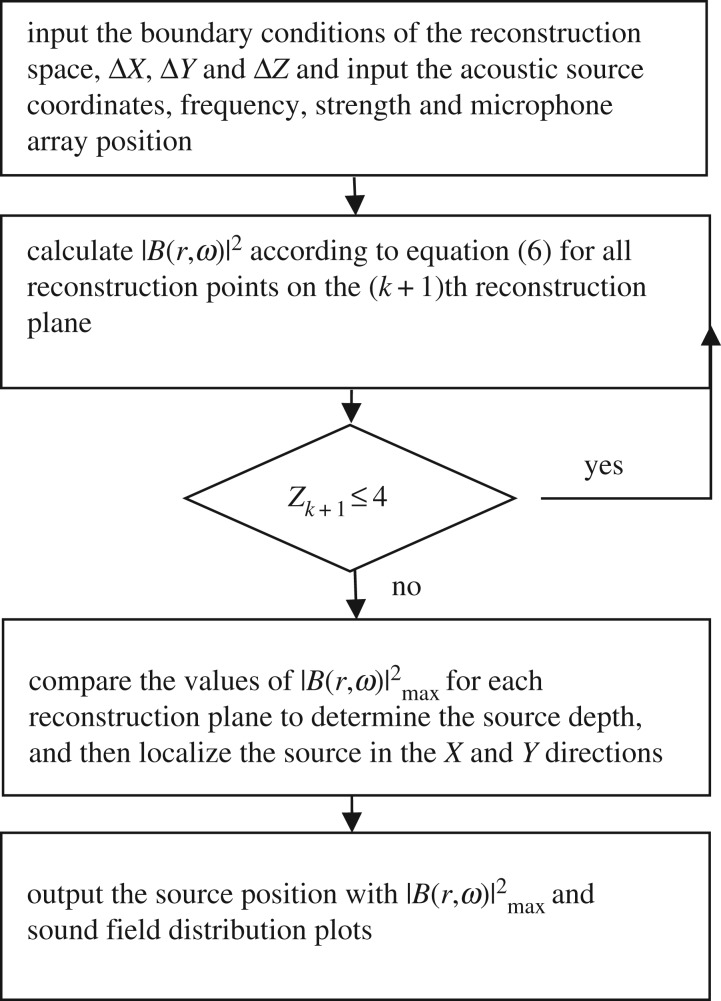
Simulation flow chart.

**Figure 3.**
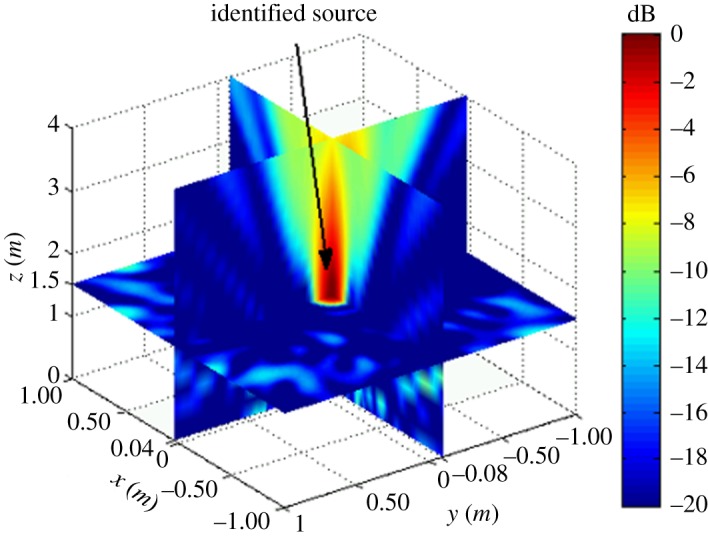
Simulation results for a single acoustic source with *x_s_* = 0.04 m, *y_s_* = −0.08 m and *z_s_* = 1.5 m on *f_s_* = 4 kHz without background noise.

**Figure 4.**
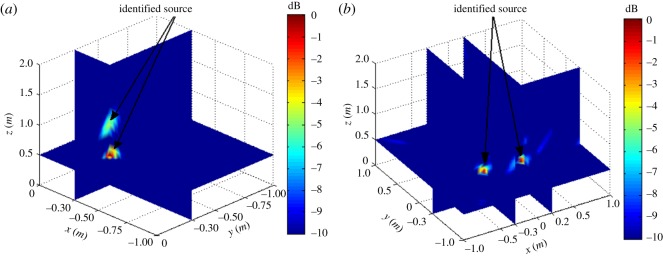
Simulation results for two acoustic sources without background noise. (*a*) *x_s_*_2_ = *x_s_*_1_ = −0.3 m, *y_s_*_2_ = *y_s_*_1_ = −0.3 m, Δ*z_s_* = *z_s_*_2_ − *z_s_*_1_ = 0.5 m, *f_s_* = 4 kHz. (*b*) *y_s_*_2_ = *y_s_*_1_ = −0.3 m, *z_s_*_2_ = *z_s_*_1_ = 0.5 m, Δ*x_s_* = *x_s_*_2_ − *x_s_*_1_ = 0.5 m, *f_s_* = 4 kHz.

The simulation assumes a rectangular reconstruction space of dimensions −1 m ≤ *x_f_* ≤ 1 m, −1 m ≤ *y_f_* ≤ 1 m and 0 m ≤ *z_k_* ≤ 4 m, where Δ*X* and Δ*Y* are 0.01 m and Δ*Z* = 0.1 m (*Z_k_*_+1_ = *Z_k_* + Δ*Z*), respectively. The assumed acoustic source is a monopole fixed in 3D space with various frequencies *f_s_*. The simulations were conducted according to the flow chart given in figure 2. To verify the possibility of the proposed localization method, two types of cases were investigated first: the single-source situation shown in figure 3 and multi-source situation shown in figure 4.

We considered four additional cases to verify the acoustic-source localization performance of the proposed method. For single-source conditions, the *X*, *Y* and *Z* positions of a source are denoted as *x_s_*, *y_s_* and *z_s_*, respectively. For multi-source conditions, the individual sources are denoted according to subscripts *s*1 and *s*2. We also consider varying values of differences in source depth between the two acoustic sources Δ*z_s_* = *z_s_*_2_ − *z_s_*_1_ and separation between the two sources in the *X* direction Δ*x_s_* = *x_s_*_2_ − *x_s_*_1_.

*Case 1:* Simulations were conducted with various source depths *z_s_* to evaluate the localization capability in the *Z* direction for a single acoustic source.
*Case 2:* Simulations were conducted with various differences in source depth Δ*z_s_* between the two acoustic sources to evaluate the localization capability in the *Z* direction under multi-source conditions.
*Case 3:* Simulations were conducted with different separations Δ*x_s_* between the two sources in the *X* direction at an equivalent source depth z_s_ to evaluate the localization capability in the *X* and *Y* directions under multi-source conditions.
*Case 4:* Simulations were conducted with equivalent depth differences Δ*z_s_* to evaluate the localization capability in the *X* and *Y* directions for different frequencies of 1.0 or 4.0 kHz under multi-source conditions.

The conditions of these cases are listed in tables 1 and 2. In the single-source scenario, the *x* coordinate of source *x_s_* is 0.04 m, the *y* coordinate of source *y_s_* is −0.08 m, the *z* coordinates of source *z_s_* are 0.5, 1.5 and 2.5 m, respectively, and source frequency *f_s_* is 1, 2.5 and 4 kHz. In the multi-source scenario, the *x* coordinate of source I *x_s_*_1_ is −0.3, −0.15, −0.1 and −0.05 m, the *y* coordinate of source I *y_s_*_1_ is −0.3 m, the *z* coordinate of source I *z_s_*_1_ is 0.5 and 1.5 m, the *x* coordinate of source II *x_s_*_2_ is −0.3, 0.2 and −0.25 m, the *y* coordinate of source II *y_s_*_2_ is −0.3 m and the *z* coordinate of source II *z_s_*_2_ is 0.5, 1 and 1.5 m. The source frequency is 1 and 4 kHz.

**Table 1.** Single acoustic-source set-up.

*x_s_* (m)	*y_s_* (m)	*z_s_* (m)	*f_s_* (kHz)		
0.04	−0.08	0.5	1.0	2.5	4.0
		1.5			
		2.5

**Table 2.** Multi-source set-up.

source	*x_s_* (m)	*y_s_* (m)	*z_s_* (m)		*f_s_* (kHz)	
source I	*x_s_*_1_	*y_s_*_1_	*z_s_*_1_		*f_s_*_1_	
	−0.3	−0.3	0.5	1.5	1.0	4.0
	−0.15					4.0
	−0.1					
	−0.05					
source II	*x_s_*_2_	*y_s_*_2_	*z_s_*_2_		*f_s_*_2_	
	−0.3	−0.3	1		1.0	4.0
	−0.3		1.5		4.0	
	0.2		0.5			
	0.25		1.5			

The planar microphone array is in the form of a 60 channel Brüel & Kjær WA-1558 sliced wheel array. The array surface is located in the *XOY* plane with its centre located at the origin. The value of $|B(r,\omega ){|}^{2}$ at all reconstruction points in 3D space was evaluated using MATLAB.

### 2.3. Experimental procedure

**Figure 5.**
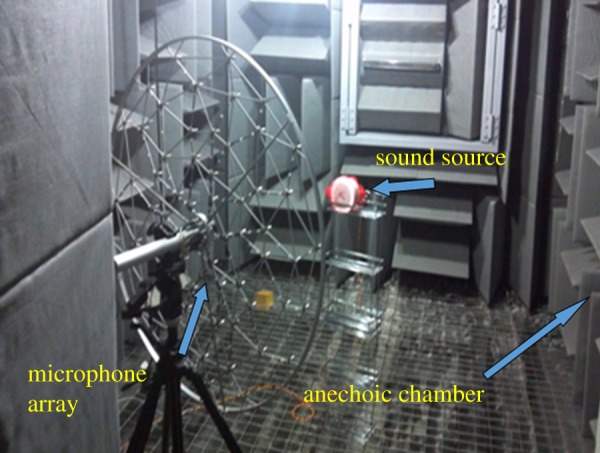
Experimental set-up.

The experiments shown in figure 5 were conducted in an anechoic chamber located at the Zhejiang University of Technology (ZJUT). The size of the chamber was 3 m × 3 m × 3 m with a background noise less than −18 dB. The minimum cut-off frequency was 63 Hz. Small speakers were employed as monopole acoustic sources. The pressure data were obtained by a 60-channel 2D Brüel & Kjær WA-1558 sliced wheel array employing acoustic imaging microphones in the chamber to verify the applicability and resolution of the proposed method. The diameter of the array is 1.05 m, and the average distance between microphones is 0.12 m. The 4958 microphone has a 10–20 kHz working frequency range, and a 28–140 dB dynamic range. The microphone layout of the array is irregular, which naturally eliminates grating lob. The array centre is set at the origin of the reference coordinate system. Each array sensor and sound source position are based on the origin of the reference coordinate system. The other settings, such as the positions of the source and array, the dimensions of the reconstruction space and the space coordinate system were equivalent to those employed in the simulation.

To evaluate the resolution of the proposed method quantitatively, we define the localization error *δ* as follows:

2.8 $$\delta =\frac{\left|\xi -\xi_{0}\right|}{\left|\xi_{0}\right|}\times 100\text{\%}.$$

where *ξ*_0_ and *ξ* are the actual and predicted *X*, *Y* or *Z* coordinates of the source, respectively. For the single-source condition, the influences of source depth and source frequency on the resolution of the proposed method were evaluated quantitatively according to the value of *δ* by changing the source depth *z*_0_ from 0.25 to 2.5 m in intervals of 0.25 m. The acoustic signal was a rectangular pulse with a strength of 30 dB and a 10 s duration, and the frequency was changed from 1 to 6 kHz in intervals of 500 Hz. For the multi-source condition, the influences of source frequency and the *X* coordinate distance of the sources on the resolution of the proposed method were evaluated quantitatively according to the value of *δ* by changing Δ*x_s_* from 0.35 to 0.8 m in intervals of 0.05 m, while *x_s_*_1_ is −0.3 m, *y_s_*_1_ is −0.3 m and *z_s_*_1_ is 0.5 m. The acoustic signals were rectangular pulses with a strength of 12 dB and a 10 s duration, and the frequency was changed from 500 Hz to 6 kHz in intervals of 500 Hz.

## 3. Results and discussion

### 3.1. Simulation results

The single-source simulation results for $|B(r,\omega ){|}^{2}$ are shown in figure 3, and the multi-source results are shown in figure 4. We note from the figures that both the single source and multi-sources can be effectively localized according to the position of $|B(r,\omega ){|}_{\max}^{2}$.

**Figure 6.**
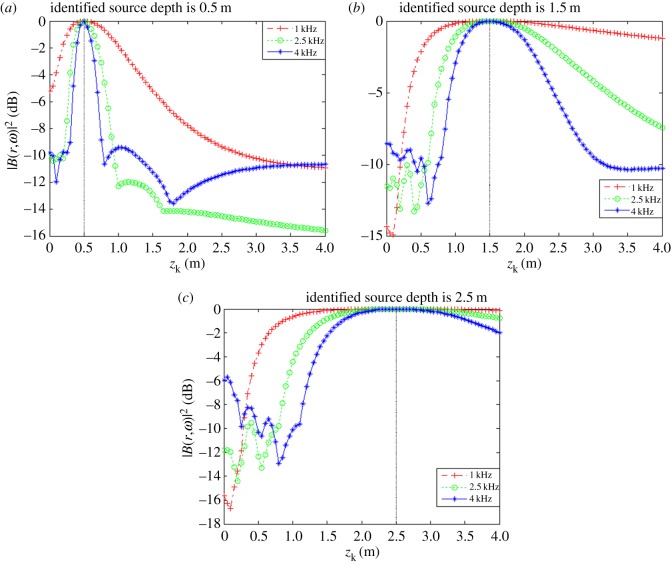
$|B(r,\omega ){|}_{\max}^{2}$ as a function of source depth *z_k_* with source frequency *f_s_*_1_ *=* 1.0 kHz, *f_s_*_2_ = 2.5 kHz and *f_s_*_3_ = 4.0 kHz. (*a*) *x_s_* = 0.04 m, *y_s_* = −0.08 m, *z_s_* = 0.5 m. (*b*) *x_s_* = 0.04 m, *y_s_* = −0.08 m, *z_s_* = 1.5 m. (*c*) *x_s_* = 0.04 m, *y_s_* = −0.08 m, *z_s_* = 2.5 m.

The obtained relationships between $|B(r,\omega ){|}_{\max}^{2}$ and *z_k_* for each value of *z*_0_ at different frequencies are shown in figure 6.

**Figure 7.**
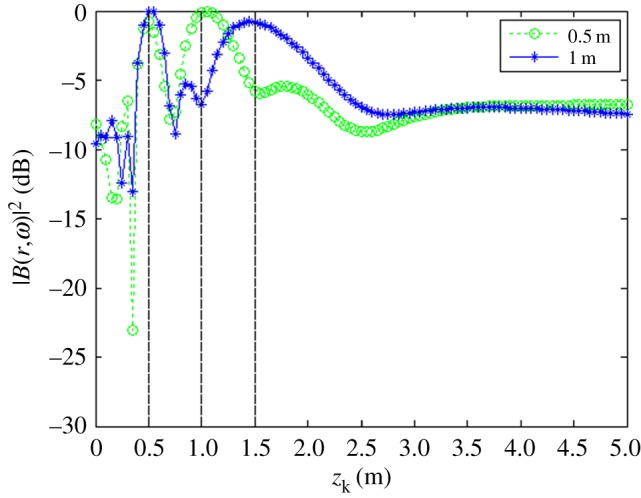
$|B(r,\omega ){|}_{\max}^{2}$ as a function of *z_k_* with Δ*z_s_*_1_ = 0.5 m and Δ*z_s_*_2_ = 1.0 m on *f_s_*_1_ = *f_s_*_2_ = 4.0 kHz.

The obtained relationships between $|B(r,\omega ){|}_{\max}^{2}$ and *z_k_* for each value of △*z_s_* are shown in figure 7.

**Figure 8.**
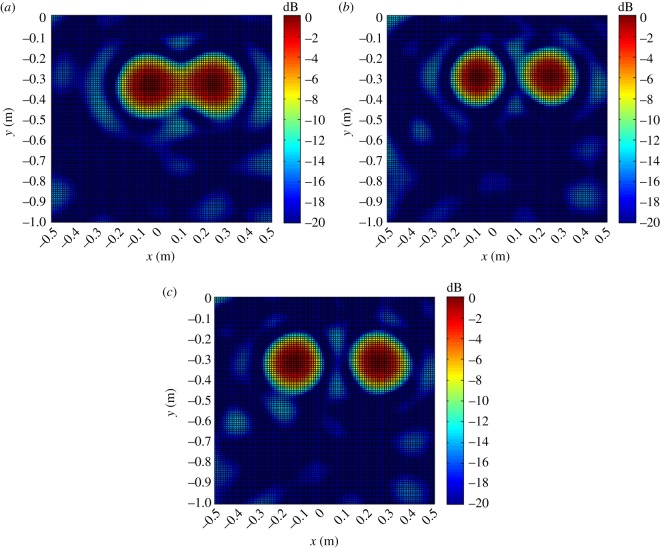
Distributions of $|B(r,\omega ){|}^{2}$ on the *XY* plane with *z_k_* = *z_s_*_1_ = *z_s_*_2_ = 1.5 m and *y_s_*_1_ = *y_s_*_2_ = *−*0.3 m on *f_s_* = *f_s_*_1_ = *f_s_*_2_ = 4 kHz. (*a*) *x_s_*_1_ = −0.05 m, *x_s_*_2_ = 0.25 m. (*b*) *x_s_*_1_ = −0.1 m, *x_s_*_2_ = 0.25 m. (*c*) *x_s_*_1_ = −0.15 m, *x_s_*_2_ = 0.25 m.

The distributions of $|B(r,\omega ){|}^{2}$ on the *XY* plane obtained at a source frequency of 4 kHz are shown in figure 8.

**Figure 9.**
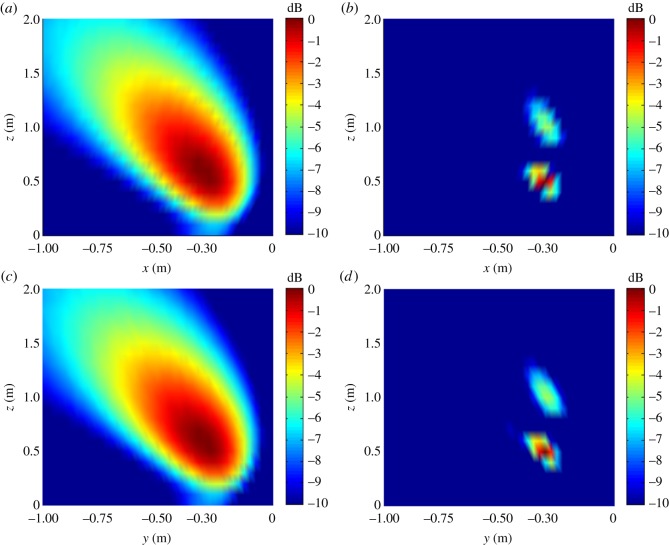
Distributions of $|B(r,\omega ){|}^{2}$ on the *XZ* or *YZ* plane with △*z_s_* = 0.5 m. (*a*) *XZ* plane, *f_s_* = 1.0 kHz; (*b*) *XZ* plane, *f_s_* = 4.0 kHz; (*c*) *YZ* plane, *f_s_* = 1.0 kHz; (*d*) *YZ* plane, *f_s_* = 4.0 kHz.

The distributions of $|B(r,\omega ){|}^{2}$ on the *XZ* or *YZ* planes are shown in figure 9.

### 3.2. Experimental results

**Figure 10.**
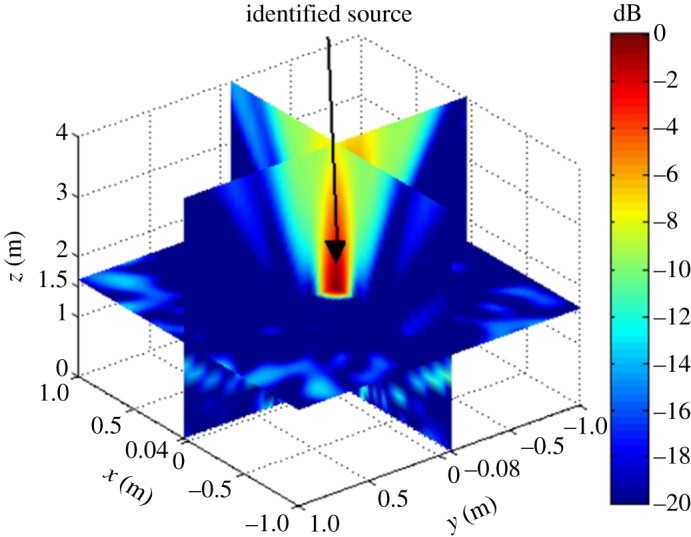
Experimental results for a single acoustic source with *x_s_* = 0.04 m, *y_s_* = −0.08 m and *z_s_* = 1.5 m on *f_s_* = 4 kHz.

**Figure 11.**
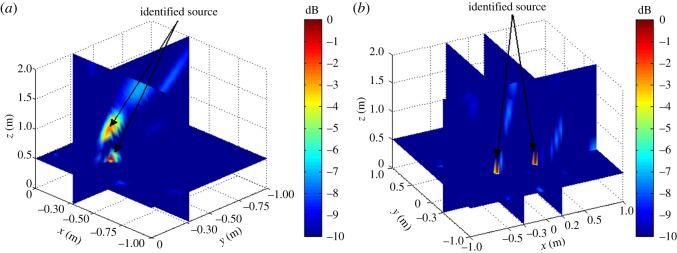
Experimental results for two acoustic sources. (*a*) *x_s_*_2_ = *x_s_*_1_ = −0.3 m, *y_s_*_2_ = *y_s_*_1_ = −0.3 m, Δ*z_s_* = 0.5 m, *f_s_* = 4 kHz. (*b*) *y_s_*_2_ = *y_s_*_1_ = −0.3 m, *z_s_*_2_ = *z_s_*_1_ = 0.5 m, Δ*x_s_* = 0.5 m, *f_s_* = 4 kHz.

**Figure 12.**
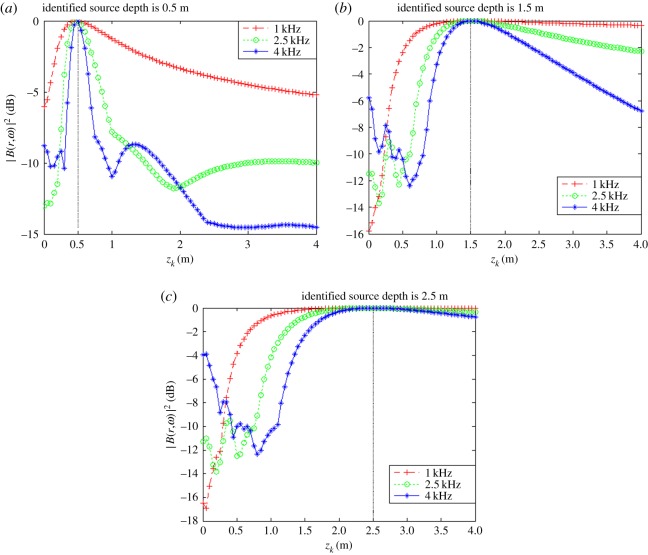
$|B(r,\omega ){|}_{\max}^{2}$ as a function of *z_k_* with *f_s_*_1_ = 1.0 kHz, *f_s_*_2_ = 2.5 kHz and *f_s_*_3_ = 4.0 kHz. (*a*) *x_s_* = 0.04 m, *y_s_* = −0.08 m, *z_s_* = 0.5 m. (*b*) *x_s_* = 0.04 m, *y_s_* = −0.08 m, *z_s_* = 1.5 m. (*c*) *x_s_* = 0.04 m, *y_s_* = −0.08 m, *z_s_* = 2.5 m.

**Figure 13.**
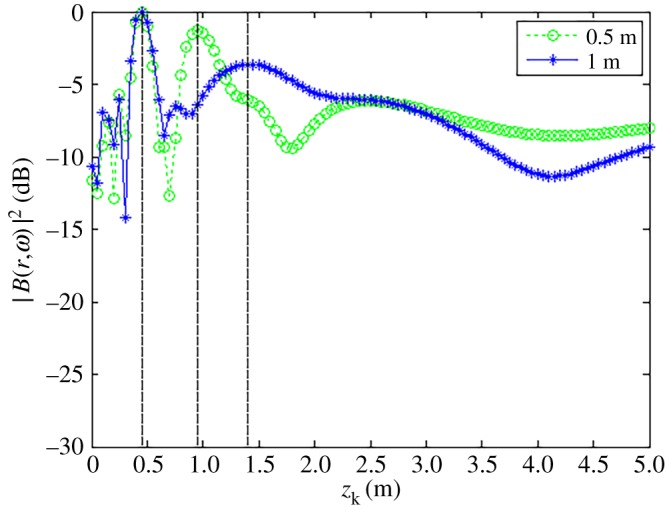
$|B(r,\omega ){|}_{\max}^{2}$ as a function of *z_k_* with Δ*z_s_*_1_ = 0.5 m and Δ*z_s_*_2_ = 1.0 m on *f_s_*_1_ = *f_s_*_2_ = 4.0 kHz.

**Figure 14.**
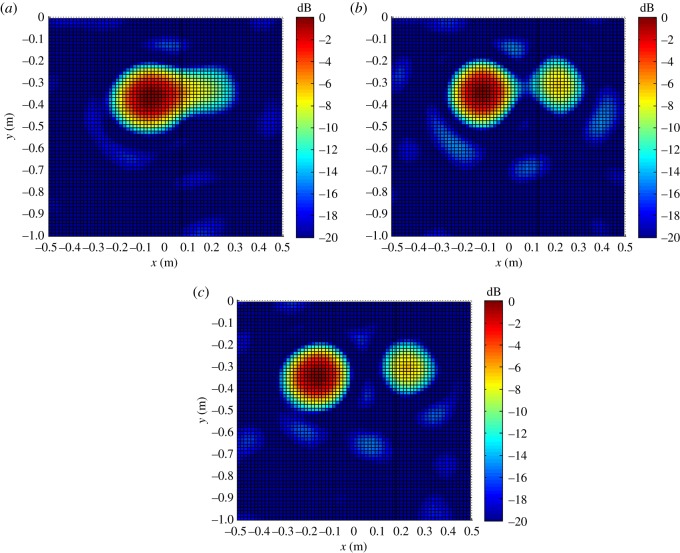
Distributions of $|B(r,\omega ){|}^{2}$ on the *XY* plane with *z_k_* = *z_s_*_1_ = *z_s_*_2_ = 1.5 m and *y_s_*_1_ = *y_s_*_2_ = *−*0.3 m on *f_s_* = *f_s_*_1_ = *f_s_*_2_ = 4 kHz. (*a*) *x_s_*_1_ = −0.05 m, *x_s_*_2_ = 0.25 m. (*b*) *x_s_*_1_ = −0.1 m, *x_s_*_2_ = 0.25 m. (*c*) *x_s_*_1_ = −0.15 m, *x_s_*_2_ = 0.25 m.

**Figure 15.**
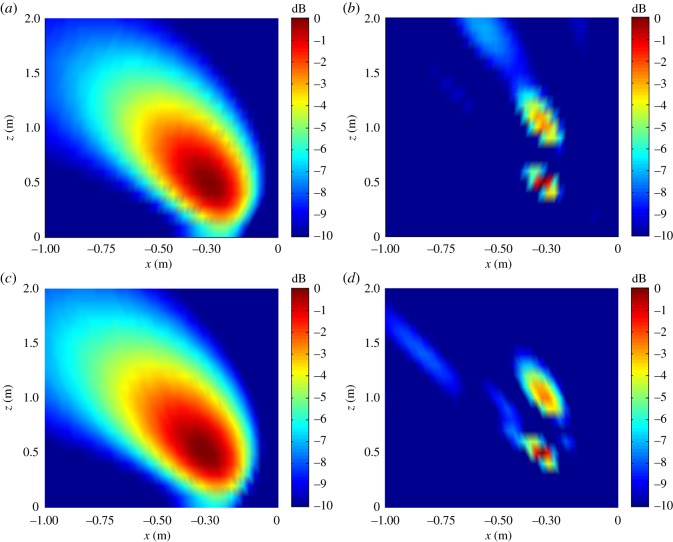
Distributions of $|B(r,\omega ){|}^{2}$ on the *XZ* or *YZ* plane with Δ*z_s_* = 0.5 m. (*a*) *XZ* plane, *f_s_* = 1.0 kHz; (*b*) *XZ* plane, *f_s_* = 4.0 kHz; (*c*) *YZ* plane, *f_s_* = 1.0 kHz; (*d*) *YZ* plane, *f_s_* = 4.0 kHz.

As discussed, the experimental conditions were equivalent to the simulation conditions to provide reliable verification of the proposed localization method. As was presented in figures 3 and 4 based on simulations, the value of $|B(r,\omega ){|}^{2}$ at all reconstruction points in 3D space obtained from the single-source and dual-source experiments is shown in figures 10 and 11, respectively. The experimentally obtained relationships between $|B(r,\omega ){|}_{\max}^{2}$ and *z_k_* presented in figures 6 and 7 based on simulations are, respectively, shown in figures 12 and 13, and the experimentally obtained distributions of $|B(r,\omega ){|}^{2}$ like those presented in figures 8 and 9 based on simulations are, respectively, shown in figures 14 and 15.

### 3.3. Discussion

In accordance with comparison of figures 4 and 10, figures 5 and 11, the simulation and experimental results indicate that the positions of $|B(r,\omega ){|}_{\max}^{2}$ are equivalent for both single-source and multi-source localization. By comparing figures 6 and 12, figures 7 and 13, the *Z* coordinates of $|B(r,\omega ){|}_{\max}^{2}$ obtained from both simulation and experiments are also equivalent. Moreover, the source can be located in the *Z* direction regardless of the source frequency or source depth. In this regard, it should be noted that the resolution in the *Z* direction is related to the distance between the array and the source, where the resolution decreases with increasing distance. In addition, the resolution in the *Z* direction is related to the value of Δ*Z*, where the resolution increases with decreasing Δ*Z*, while decreasing Δ*Z* also increases the computational burden of the method, resulting in an increasing computational time. Comparing figures 8 and 14, the simulation and experimental results provide equivalent *X* and *Y* coordinate positions for $|B(r,\omega ){|}_{\max}^{2}$, where *zk* is equal to *z*0. This indicates that the proposed method can locate the source along the *X* and *Y* directions after determining the position along the *Z* direction. Therefore, the concept of the recognition direction must be derived, and the optimum condition of the surface position of the array is parallel to the measurement surface. Under this condition, these conclusions are applicable to the whole search area. The resolution for the *X* and *Y* directions is related to the distance between the array and the source in an equivalent manner as was discussed for the *Z* direction. Comparing figures 9 and 15, both the simulation and experimental results indicate that the resolution along the *X*, *Y* and *Z* directions is significantly related to the source frequency.

**Figure 16.**
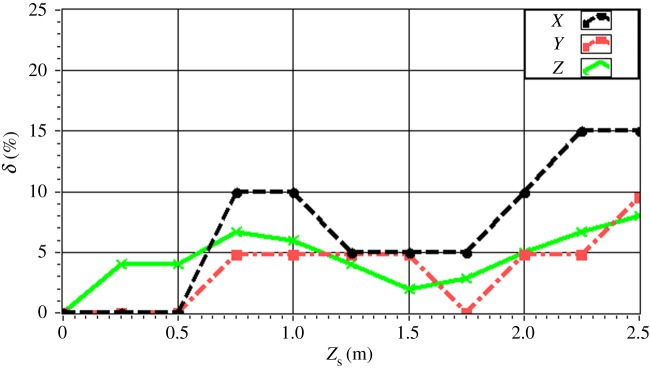
Experimental localization errors *δ* in the *X*, *Y* and *Z* directions as a function of single source depth *z_s_* for *f_s_* = 4.0 kHz.

**Figure 17.**
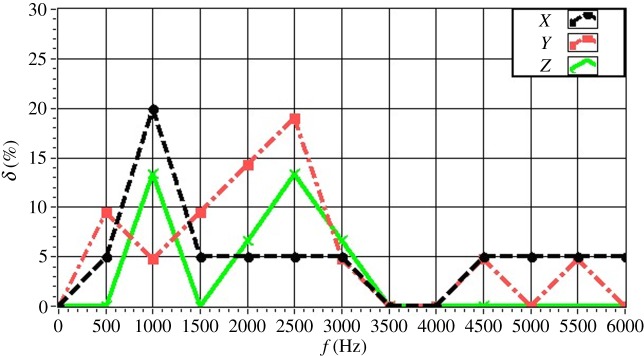
Experimental values of *δ* in the *X*, *Y* and *Z* directions as a function of *f_s_* for *z_s_* = 1.5 m.

**Figure 18.**
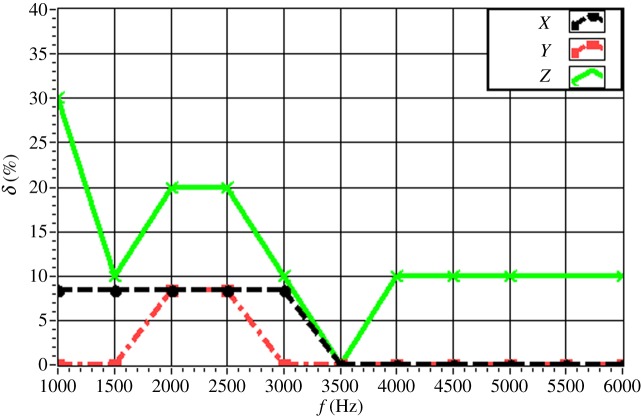
Experimental values of *δ* with respect to *f_s_* in the *X*, *Y* and *Z* directions for two sources (source I) with Δ*z_s_* = 1.0 m.

**Figure 19.**
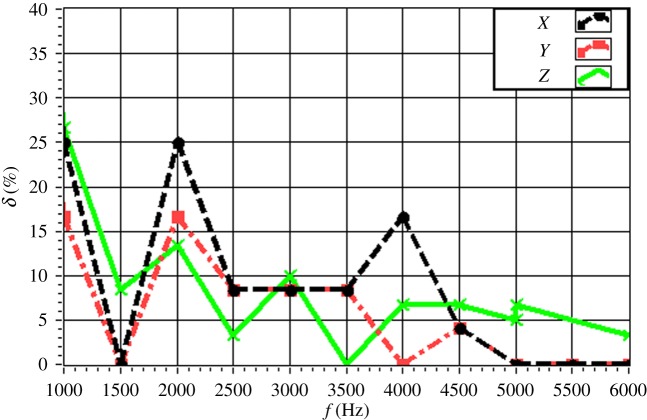
Experimental values of *δ* with respect to *f_s_* in the *X*, *Y* and *Z* directions for two sources (source II) with Δ*z_s_* = 1.0 m.

**Figure 20.**
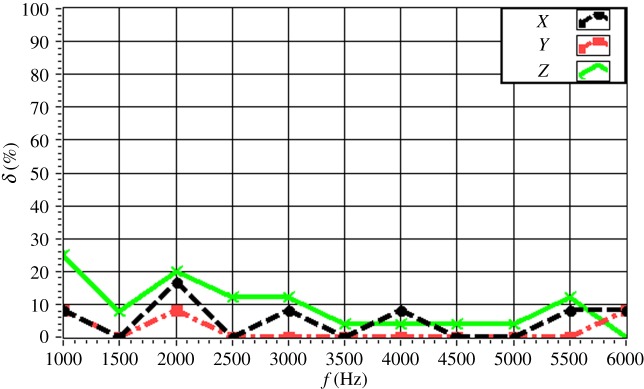
Experimental values of *δ* with respect to *f_s_* in the *X*, *Y* and *Z* directions for source I with Δ*x_s_* = 0.5 m.

**Figure 21.**
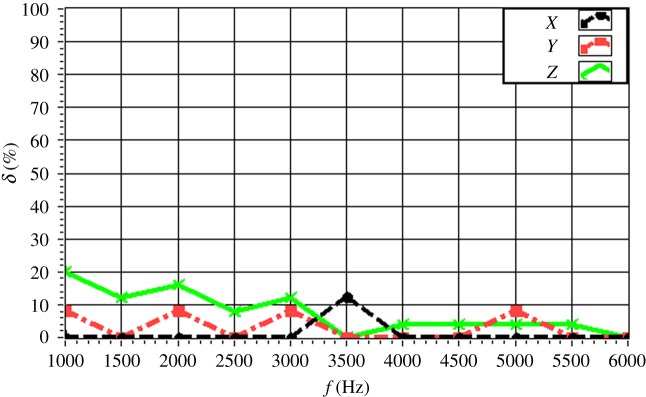
Experimental values of *δ* with respect to *f_s_* in the *X*, *Y* and *Z* directions for source II with Δ*x_s_* = 0.5 m.

**Figure 22.**
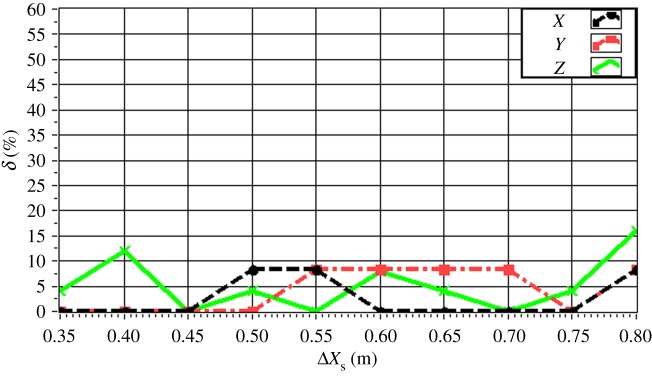
Experimental values of *δ* with respect to Δ*x_s_* in the *X*, *Y* and *Z* directions for source I with *f_s_* = 4.0 kHz.

**Figure 23.**
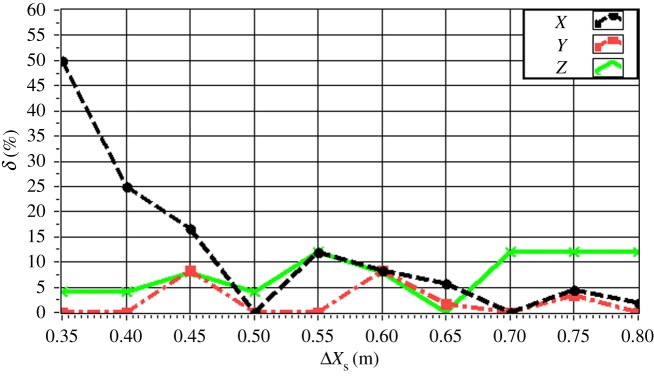
Experimental values of *δ* with respect to Δ*x_s_* in the *X*, *Y* and *Z* directions for source II with *f_s_* = 4.0 kHz.

The experimentally obtained values of *δ* for a single source (*f* = 4.0 kHz) are given in figure 16 with respect to source depth. The value of *δ* is maintained within 15% in all directions. In particular, the value of *δ* is less than 10% for a source depth less than 2 m. The value of *δ* increases with increasing source depth, which reflects the discussed decrease in the resolution with increasing source depth owing to the increasing distance between the source and the array. The experimentally obtained values of *δ* for a single source (*z_s_* = 1.5 m) are given in figure 17 with respect to source frequency. The value of *δ* is maintained within 20% in all directions. In particular, the value of *δ* is less than 10% for a frequency greater than 3.0 kHz. The value of *δ* decreases with increasing frequency, which reflects an increasing resolution with increasing source frequency. The experimentally obtained values of *δ* for two sources (Δ*z_s_* = 1.0 m) with respect to source frequency are given in figures 18 and 19 for source I and source II (table 2), respectively. Under these conditions, we found that the values of *δ* in the *X*, *Y* and *Z* directions for source I and source II fluctuate within 20% for frequencies greater than 1.5 kHz. However, with increasing frequency to 4.0 kHz, the values of *δ* fluctuate within 10%. Thus, the fluctuations in *δ* flatten out, and *δ* decreases with increasing source frequency. Similarly, the experimentally obtained values of *δ* for two sources (Δ*z_s_* = 0.5 m) with respect to source frequency given in figures 20 and 21 for source I and source II, respectively, show that the values of *δ* in the *X*, *Y* and *Z* directions fluctuate within 20% for frequencies greater than 1.5 kHz. However, the values of *δ* again fluctuate within 10% with increasing frequency to 4.0 kHz, representing a flattening of fluctuations and a decreasing *δ* with increasing source frequency. By contrast, the experimentally obtained values of *δ* for two sources (*f_s_* = 4.0 kHz) with respect to Δ*x_s_* given in figures 22 and 23 show that, for source I, the values of *δ* in the *X*, *Y* and *Z* directions fluctuate within 15% at small values of Δ*x_s_*, and the fluctuations in *δ* for source II decrease with increasing Δ*x_s_*, eventually maintained at a value within 15% for Δ*x_s_* greater than 0.5 m.

## 4. Conclusion

This paper presented a BBAI method to locate acoustic sources using a planar microphone array. The proposed BBAI method was evaluated by simulations and physical experiments in an anechoic chamber employing single and multiple monopole acoustic sources. The results obtained from both simulations and experiments demonstrated that the proposed method can effectively locate monopole sources in 3D. Based on the obtained results, we can conclude that the localization error (*δ*) in the *X*, *Y* and *Z* directions increases with increasing source depth (*z*_0_) for a single acoustic source or with increasing difference between source depths (Δ*z_s_*) for two acoustic sources, and the spatial resolution correspondingly decreases. Good spatial resolution can be expected for a source depth less than 2.0 m. In addition, the values of *δ* tended to decrease with increasing source frequency (*f_s_*), particularly for *f_s_* greater than 3.0 kHz, resulting in increased spatial resolution with increasing *f_s_*. Furthermore, the values of *δ* tended to decrease as the interval between the *X* coordinates of the acoustic sources (Δ*x_s_*) increased, and *δ* was maintained within 15% for Δ*x_s_* greater than 0.5 m. Moreover, fluctuations in *δ* flattened and *δ* decreased under these conditions with increasing *f_s_*. However, the fact that the localization precision declined with increasing Δ*z_s_* under multi-source localization indicates that the proposed BBAI method includes some limitations that must be addressed. Therefore, the proposed method should be subjected to further development in terms of several aspects, such as regarding multi-source identification and parameter optimization in terms of the shape and size of the focusing plane or the mesh size employed in the analysis. In addition to the influence of measurement noise, the influences of array sensor installation errors, confusion error and other measurement errors [31,32,42] should also be considered. We hope that this method can be used in more industrial applications. And next, we will try new research in the field of medical ultrasound [8] and underwater acoustic sensor [43,44].

## Data accessibility

This article does not contain any additional data.
